# Teachers at risk: Depressive symptoms, emotional intelligence, and burnout during COVID-19

**DOI:** 10.3389/fpubh.2023.1092839

**Published:** 2023-03-09

**Authors:** Laura Sánchez-Pujalte, Talía Gómez Yepes, Edgardo Etchezahar, Diego Navarro Mateu

**Affiliations:** ^1^Faculty of Education, International University of Valencia, Valencia, Spain; ^2^Department of Inclusive Education, Faculty of Education, Catholic University of Valencia, Valencia, Spain; ^3^Faculty of Psychology, University of Buenos Aires, Buenos Aires, Argentina; ^4^National Scientific and Technical Research Council, Buenos Aires, Argentina

**Keywords:** teachers wellbeing, depressive symptoms, emotional intelligence, burnout, COVID-19

## Abstract

**Background:**

Previous studies indicated that depressive symptoms are common among teachers due to job stress and difficulty in managing emotions. The aim of this research was to determine the levels of depressive symptomatology in a sample of secondary school teachers who worked during the COVID-19 pandemic and to analyze the relationships with their levels of burnout and emotional intelligence.

**Methods:**

The study involved 430 secondary school teachers residing in Madrid (Spain) who worked during the COVID-19 pandemic. Participants' age was between 25 and 60 (*M* = 41.40; SD = 11.07) and the gender distribution was 53.72% men and 46.28% women. We used the Spanish version of the Patient Health Questionnaire (PHQ-9), the Maslach Burnout Inventory Educators Survey (MBI-ES) and the Trait Meta-Mood Scale (TMMS-24).

**Results:**

The main results indicated that teachers presented high means of depressive symptomatology, with women obtaining higher scores than men. Significant relationships were also observed between the levels of depressive symptomatology and the dimensions of burnout and emotional intelligence. Finally, the three dimensions of emotional intelligence would contribute to the depressive symptomatology of teachers, while of the burnout dimensions only Emotional Exhaustion would make a contribution.

**Conclusion:**

The possible consequences of depressive symptomatology in teachers during the pandemic are discussed, as well as the need to enhance protective factors such as emotional intelligence and to study burnout levels.

## Introduction

The COVID-19 pandemic, one of the greatest challenges faced by humanity this century, has had negative consequences on the psychosocial wellbeing of the world's population (1–3). Multiple studies highlight that one of the population groups that was most affected by the appearance or increase of symptoms that could be associated with stress is the teachers one (4–6). Different researches carried out during the last years confirms that, within the multiplicity of existing professional groups, teachers are one of the most exposed to occupational stress (7–9) and to different common mental disorders associated with it (10, 11). One aspect that characterizes teaching, and is shared by health and social care professions, is the high level of “work emotion” required (4, 12, 13). That is, workers are expected to manage their feelings according to the norms and guidelines defined by the organization, place and type of work (14, 15). In the particular case of teachers, a large part of their work involves direct interaction with students and their families or legal representatives, an action that requires an adequate emotional education to be able to manage and express emotions diligently during the different encounters (16). Such contextual conditions coupled with unsustainable work environments, as well as personal characteristics (17), can make teaching challenging and lead to prolonged episodes of stress and physical and emotional exhaustion (7, 18).

Teaching is one of the professions in which there are high levels of sick leave. Teachers are vulnerable to physical ailments, such as headaches, but also to psychological conditions such as anxiety, stress and depressive symptoms (19). Such conditions can lead to sick leave, decreased professional commitment and absenteeism as a result of prolonged stress (20, 21). In the case of Spain, figures from the Confederación Salud Mental España (CSME) (22) show that one of the most affected professional fields is the educational one, with a higher prevalence of stress, anxiety and depressive symptomatology caused or aggravated by work. According to the CSME report (22), 90% of the teaching staff suffered sleep disturbances due to the pandemic, 89.5% feel more irritable and 42.7% feel depressed or unhappy at work. In the same line, a study carried out by Affor Prevención Psicosocial (23), 54% of teachers show symptoms of anxiety and depression caused by COVID-19 and the return to the classroom. According to the consultancy firm, the impact of COVID-19 has caused psychological imbalances in the working population, as they are immersed in an unknown and hostile environment such as the one generated by the pandemic. In addition, the education sector has traditionally been one of the most exposed to pathologies associated with the workplace. The COVID-19 pandemic highlighted the need to develop sustainable policies and work environments that provide wellbeing to teachers in different aspects (7), which would be reflected both in higher performance—even in borderline scenarios—, as in the case of job performance during the COVID-19 pandemic (3, 24, 25). It would also be reflected in the reduction of physical ailments such as sore throat or headache for which teachers are part of the vulnerable working population, as well as mental health problems (9) that can cause sick leave, disengagement and even absenteeism as some of its consequences (21). With the start of a new school year, education departments and schools should reinforce the monitoring of psychosocial risks and mental health problems associated with prolonged stress (7, 26) among teachers, to avoid its persistence over time. This phenomenon is known as Burnout—recently recognized as a disease by the World Health Organization (27) in the 11th revision of the International Classification of Diseases (ICD-11) (28)—and its incidence can lead not only to a decrease in the effectiveness of professionals, but also to depressive symptoms (10).

Different studies and theoretical approaches developed during the last decades (29, 30) show how different crises, regardless of whether they are economic (31), health (5), war (32–34), etc., affect people's mental health (35). In Spain, the recent health crisis due to COVID-19 seems to have negatively affected the mental health of its residents, generating or increasing depressive symptomatology-particularly secondary school teachers. According to CSME reports (22), this group of professionals would require a more accurate assessment and diagnoses for adequate care and intervention, due to depression is one of the main causes of disability.

International guidelines recommend the assessment of depressive symptomatology from primary care centers (36), however, some authors also raise concerns about mass assessment, not only because of the overestimation of symptomatology, but also because of the number of existing assessments (37). In this regard, one of the most reliable assessments is the Patient Health Questionnaire (PHQ-9) (38–40), a tool that makes it possible to establish a provisional diagnosis of major depressive disorder on the basis of the answers to the questions in the questionnaire.. According to the Diagnostic and Statistical Manual of Mental Disorders (41), major depressive disorder is likely to be present if at least five of the nine symptoms “during the same period within 2 weeks and represent a change from previous functioning.” At least one of the symptoms is (1) depressed mood or (2) loss of interest or pleasure (41) in performing activities (questions 1 and 2 of the PHQ-9)—suicidal ideation should also be taken into account in this criterion. The symptomatology should also lead to elevated distress and loss of operability. Also, symptoms should not be further defined by substance use or another medical or psychiatric condition. “Other” depression is diagnosed if there is significant wasting or discomfort in core areas of functioning, but not all criteria for any specific depressive disorder are met. While the PHQ-9 can be used to diagnose major depressive syndrome, additional information is also required on the occurrence of manic episodes, other mental disorder, medication side effects, among others (39).

Another ailment that most afflicts teachers is prolonged stress or burnout. Burnout syndrome is defined (21) as a persistent negative emotional state, which is characterized by low self-esteem, decreased motivation and professional commitment, and generalized psychological discomfort. This is a consequence of the long-term stress caused by the working environment (42). Burnout syndrome is characterized by three dimensions: depersonalization, Emotional Exhaustion and reduced personal accomplishment. The first dimension refers to an apathetic or numb response to professional commitments (43). Emotional exhaustion refers to extreme feelings of emotional and physical exhaustion. The last dimension refers to the worker's self-perception of his or her ability to cope with professional challenges (2, 42), which can lead to feelings of failure, worthlessness and low self-esteem.

Studies on burnout syndrome in teachers determine the convergence of different factors related to individual differences (4, 14) and emotional exhaustion, caused by the increase and level of stressors—as may be the case of increased workload—(44, 45). In the educational setting, teachers may go through periods of burnout due to changes in their professional conditions—as is the case of the change of circumstances due to the COVID-19 pandemic—(46). Teachers experienced the increase or appearance of stressors such as decreased professional autonomy (9, 47), interpersonal disputes with co-workers and relatives (5, 48) due to the inability to distinguish work hours from leisure or rest hours (49), among others. Such changes can lead to fluctuations in motivation, causing burnout and decreasing the capacity to regulate internal emotional reactions (50), leading over time to the appearance of depressive symptomatology (51). However, not all people present symptomatology at the same level (52, 53). What happens so that under the same working conditions some individuals suffer extreme stress or present depressive symptomatology and others do not? It is clear that not all people respond in the same way (54). The evaluation that each person makes to respond to the same events could be influenced, among other factors, by emotional intelligence as a skill, which would function as a coping mechanism, a protective factor (55) that allows people to develop resources to protect themselves from deviant behavior (56–58). Therefore, the lack or poor development of emotional intelligence could generate or increase vulnerability to the consequences of Burnout (59, 60).

As a coping mechanism against the negative effects of burnout, emotional intelligence has been reported to have a high effect on people's ability to cope with it (59, 61). Emotional intelligence is the collection of skills involved in the processing of emotions and affective information (62–65), which are divided into three major dimensions: emotional clarity, mindfulness and repair (66). On the one hand, *Emotional Clarity* has to do with the way in which people perceive their emotions. Teachers' unstable moods would be related to low levels in this dimension. Such moods would lead to harmful thoughts and result in situations of significant stress (59, 63, 67). The psychological inability to modify emotional states would result in negative consequences at the work level (18, 42), such as inappropriate teaching practice, burnout or abandonment, as psychosocial wellbeing would be affected (50, 63). On the other hand, *Attention* dimension refers to the degree of attention that people believe they pay to their moods. Being affected would mean a decrease in performance and an increase in distracters, leading teachers to neglect daily tasks. The third dimension is *Repair*, or people's belief in their ability to regulate their emotional states, prolonging positive ones and interrupting negative ones. When this dimension is affected in teachers, there is a decrease in the strategies and capacity to regulate stress, leading to burnout (59, 68). The repair dimension, at high levels, would lead to intellectual and emotional growth of teachers. High levels in the three dimensions would function as a protective measure against burnout experienced by teachers in stressful and complex situations, as in the case of the COVID-19 pandemic (21, 59, 66).

Given that teaching-specific stressors are mainly related to emotional factors (69), and the significant increase in interventions aimed at teachers' emotional learning in recent years (49, 59, 70, 71), an investigation was carried out to find out the levels of depressive symptoms in secondary school teachers associated with prolonged stress caused by the COVID-19 pandemic containment. The role of emotional intelligence as a regulatory mechanism or protective shield against the appearance or increase of such symptomatology was investigated.

The aim of this work was to analyze whether teachers felt that their work performance was affected by the COVID-19 pandemic, to know their depressive severity indexes and to analyze their levels of stress and emotional intelligence as possible obstructive-protective factors.

In this sense, the following research hypotheses are proposed: H1. Teachers' levels of depressive symptoms are medium; H2. There are differences in depressive symptomatology according to gender, but not according to the age and years of experience of the teachers, H3. Depressive symptomatology is related to the burnout and emotional intelligence dimensions. H4. The burnout and emotional intelligence dimensions (Attention, Clarity and Repair) influenced participants' depressive symptomatology.

## Materials and methods

### Participants

We conducted a cross-sectional study, with a sample of 430 secondary school teachers from Madrid (Spain) who worked during the COVID-19 Pandemic in 2020/2021. The age of the participants was 25–60 years (*M* = 41.40; SD = 11.07) and the distribution on the gender was 231 males (53.72%), and 199 (46.28%) females.

### Measures

#### Patient Health Questionnaire (PHQ-9)

The Spanish version of the Patient Health Questionnaire scale was used, which consists of nine items that evaluate the presence of depressive symptoms present in the last 2 weeks. The items has a severity index corresponding to: 0 = “never,” 1 = “some days,” 2 = “more than half of the days” and 3 = “almost every day.” According to the scores obtained on the scale, the following classification is obtained: (1) Normal (scores from 0 to 4), (2) Mild (scores from 5 to 9), (3) Moderate (scores from 10 to 14), and (4) The reliability reported by the Cronbach's alpha for the instrument was adequate (*α* = 0.88).

#### Maslach Burnout Inventory Educators Survey

Burnout levels were evaluated with the Maslach Burnout Inventory Educators Survey (MBI-ES) adapted for teachers (19). The MBI-ES has demonstrated adequate reliability and validity of the three-factor structure in different research reports. Cronbach's alpha for the MBI-ES scales ranged from 0.88 to 0.90 for Emotional Exhaustion, 0.74 to 0.76 for Depersonalization, and 0.72 to 0.76 for Personal Accomplishment. Adequate convergent and discriminant validity of the original MBI was established as the measure was developed (72). The scale has 22 items with answer choices on a six-point Likert-type scale (from 0 = never to 6 = always/every day), on three scales: Emotional Exhaustion (feeling of not being able to do more, finding oneself physically and emotionally exhausted), Depersonalization (an unfeeling and impersonal response toward recipients of one's instruction), and Personal Accomplishment (feelings of competence and successful achievement in one's work). The reliability reported by the Cronbach's alpha for the three dimensions was adequate (0.77 ≤ *α* ≤ 0.82).

#### Emotional intelligence (TMMS-24)

We use the TMMS-24, the Spanish version (62) of the TMMS-48 (63, 64). The TMMS-24 is original composed of 24 items that make up three dimensions: Attention, Emotional Clarity and Emotional Regulation, with a five-point response format Likert-type scale (1 = strongly disagree, to 5 = strongly agree). The original Spanish version of the scale analyses the descriptive statistics of the items, and the internal consistency of the three dimensions (0.77 < *α* < 0.82), as well as the construct validity was adequate. Regarding the psychometric properties of our study, we observed an adequate reliability (Attention: *α* = 0.80; Clarity: *α* = 0.79 and Regulation: *α* = 0.78).

### Sociodemographic data

We use an *ad-hoc* questionnaire to ask age, gender, and years of experience as a teacher.

### Procedure

The data was collected on an online survey delivered *via* social media to several groups of teachers in Madrid (Spain). The inclusion/exclusion criteria for being able to participate in our study was that the teacher had given classes in secondary schools during 2020 and 2021. Before completing the questionnaire, teachers were provided with the appropriate instructions and their participation was optional and anonymous. The average time estimated to complete the questionnaire was between 10–15 min.

### Data analysis

The statistical analysis was carried out with SPSS 24 (73). First, to test H1, the descriptive statistics of teachers' PHQ9 levels were analyzed (frequencies and percentages). Subsequently, to test the H2 about gender differences in depressive symptomatology a set of student's *t*-test was applied (as well as the Cohen's *d* for effect size). Likewise, Person's correlations were used to study the relationships between age and years of teaching experience of the participants with their depressive symptomatology. Regarding H3, a series of Pearson's correlations was conducted to find out whether there were differences between PHQ9, the burnout dimensions according and emotional intelligence dimensions. Finally, linear regressions were used to analyse the extent to which burnout and emotional intelligence dimensions (Attention, Clarity, and Repair) influenced participants' PHQ9 levels. In all cases, the normality criteria and the outliers of the structured variables were calculated to perform the parametric analyses.

## Results

First, we analyzed the extent to which teachers felt that their work performance was affected by the COVID-19 pandemic. Only 9% of the participants indicated that they had not been affected, while 29% reported feeling somewhat affected, 44% quite affected, and 18% very affected.

According to the PHQ9 results, Table 1 shows the percentages of teachers who do not have symptoms of severity and those who have mild depression. Likewise, when adding the percentages of moderate, moderate to severe and severe depression, 16.2% is observed (Table 1).

**Table 1:** Level of severity of PHQ9 in the sample of teachers.

**Degree of severity**	**Range**	**%**
Normal	0–4	44.1
Mild	5–9	39.7
Moderate	10–14	6.7
Moderate to severe	15–19	7.3
Severe	20–27	2.2
**Total**		100.0

Female teachers (*M* = 1.96; *SD* = 1.04) had significantly more depressive symptomatology (*t* = 2.275; *p* < 0.05; Cohen's *d* = 0.35) than male teachers (*M* = 1.62; *SD* = 0.88). No differences were observed according to the age and years of teaching experience of the participants with respect to their depressive symptomatology.

Subsequently, we proceeded to analyze the relationships between the levels of depressive severity, burnout and emotional intelligence of the participants (Table 2).

**Table 2:** Relationships between PHQ9, burnout, and emotional intelligence.

	**1**	**2**	**3**	**4**	**5**	**6**	**7**
1. PHQ9	–	0.086	−0.323^**^	−0.438^**^	0.525^**^	−0.287^**^	0.275^**^
2. TMMS attention		0.801	0.414^**^	0.367^**^	0.132	−0.050	0.007
3. TMMS clarity			0.887	0.533^**^	−0.259^**^	0.151	−0.349^**^
4. TMMS repair				0.825	−0.187^*^	0.254^**^	−0.155
5. Emotional exhaustion					0.930	−0.315^**^	0.509^**^
6. Depersonalization						0.832	−0.211^**^
7. Personal accomplishment							0.780

Cronbach's Alfa in the diagonal.

* p < 0.01.

** p < 0.001.

As can be seen in Table 2, the levels of depressive severity ascertained through the PHQ are significantly related to the Clarity and Repair dimensions of emotional intelligence. That is, the higher the levels of these two dimensions of emotional intelligence, the lower the level of depression. Conversely, the higher the levels of the three dimensions of burnout, the higher the levels of depressive symptomatology, particularly the levels of Emotional Exhaustion, which has a medium-high strength.

Finally, the contribution of the dimensions of emotional intelligence and burnout on the levels of depressive severity was analyzed through a series of regressions (Table 3).

**Table 3:** Regression model results for the PHQ9.

**Step**	**Predictor**	**β**	***R* ^2^**	**Δ*R*^2^**
1	TMMS attention	−0.359^***^	0.242	0.259^***^
	TMMS clarity	−0.285^**^		
	TMMS repair	−0.345^***^		
2	TMMS attention	0.231^***^	0.374	0.143^**^
	TMMS clarity	−0.187^*^		
	TMMS repair	−0.262^***^		
	Emotional exhaustion	0.403^***^		
	Depersonalization	−0.075		
	Personal accomplishment	−0.047		

** p < 0.01.

*** p < 0.001. The asterisk represents statistically significant differences.

The three dimensions of emotional intelligence contribute to the levels of depressive severity, as can be seen in Table 3. With respect to the burnout dimensions, the only one that makes a significant contribution is Emotional Exhaustion, while depersonalization and Personal Accomplishment do not contribute to the PHQ9 levels.

## Discussion and conclusions

Although an important part of the participants indicated that they had been significantly affected emotionally by the pandemic (91% indicated having been affected between a little and a lot), this percentage decreased in terms of the severity of depression assessed by the PHQ-9, since 39.7% presented indicators of mild depression, while 16.2% were between moderate and severe (the sum of both percentages results in 51.9%, also a significant number). This may be due to the *ad hoc* overestimation and generalization of depressive symptomatology by the participants. Most people experience transitory mood fluctuations, however, the pandemic brought with it extreme situations never before experienced by human beings (2, 4, 5, 9). Some authors (37) also posit that the overestimation of symptomatology could be due to the massive assessment, and also to the number of existing assessments.

Also, women presented higher levels of depressive symptomatology compared to men. These results are consistent with previous studies showing a higher incidence of depressive disorders in women than in men (74). These gender differences could be partly explained by women's greater exposure to adverse situations, risk factors within which include violence against women, structural gender inequality, among others (75). These risk factors were enhanced by the isolation decreed by the COVID-19 pandemic. According to multiple studies carried out during and post pandemic, the circumstances of confinement would have potentiated daily stressors, with women being the group most affected by health, work and personal measures both globally (76, 77) and in the case of Spain (78, 79). No differences were observed in other demographics (age, years of experience in teaching practice).

Regarding the relationships between the PHQ9 and the dimensions of emotional intelligence, negative relationships were observed with the Clarity and Repair dimensions, i.e., the higher the levels in these dimensions, the lower the depressive severity assessed through the PHQ9. No differences were observed with the Attention dimension. Regarding the relationships between the PHQ9 and the burnout dimensions, significant and positive relationships were observed with Emotional Exhaustion and Personal Accomplishment (i.e., the higher the levels of this variable, the greater the depressive severity) and negative relationships with Depersonalization (the higher the levels of these variables, the lower the levels of depressive severity). Taking into account the above information and results, and with the assumption that work environment is one of the main factors to be taken into account for the development of psychologically healthier teachers (7, 18), the promotion of teachers' wellbeing becomes a key factor for their own productivity and sustainability (3), helping to prevent pathologies like burnout and depressive symptoms associated with work and teaching environments (42). Much remains to be investigated and explored after a pandemic such as the one the whole world has experienced, although the efficacy of these protective factors has been extensively studied.

Regarding burnout, of the three dimensions (Emotional Exhaustion, Personal Accomplishment, and Depersonalization) only Emotional Exhaustion was significant. These results are consistent with previous studies on burnout symptoms (51, 80, 81) that showed that depressive symptomatology correlated more strongly with emotional exhaustion (82) considered the central dimension of the MBI-ES.

Finally, when we testing to what extent the dimensions of burnout and emotional intelligence contributed to the levels of depressive severity, it was observed that the three dimensions of emotional intelligence made a significant contribution. These results are consistent with previous research findings which showed that a high development of emotional skills could be considered a valuable resource in the prevention of mental illness (5, 83). This implies that emotional intelligence would function as a protective factor, indicating the need for training in each of its dimensions.

Taking into account previous literature and the results of this study, it is concluded that it is important for teachers to have training in emotional intelligence (10, 55), not only to be able to manage their own emotions, but also to provide support for the resolution of similar problems, both to the rest of the teaching staff and to the students (18). In this way, the knowledge, attitudes, training and educational models from which teachers work come into play (4, 5). With a view to the future, it is necessary to include in teacher training plans a perspective that includes instruction in emotional education and in the recognition of symptoms associated with exposure and situations of prolonged stress (84, 85). In this way, it will be possible to prevent and deal with different forms of socio-labor exclusion that teachers suffer in the educational context as a consequence of burnout (19). Thus, education must participate at the institutional and professional level as an engine for transformation and improvement in terms of education and public health, seeking to achieve more inclusive and safer future societies for teachers and by extension for students.

## Limitations

One of the main limitations of this study is that since it was a cross-sectional study, it was not possible to examine the trajectory of depressive symptomatology, nor the symptoms of Burnout. Another limitation would also be the size of the sample (72). Although the size of the sample allowed us to corroborate the hypotheses, it is not large enough, an aspect that will be taken into consideration for future studies. Therefore, it was not possible to analyze with certainty whether the depressive symptomatology observed in the participants is a product of burnout or vice versa. It is also uncertain whether the Burnout symptoms are a consequence of the change in working conditions due to the confinement decreed by the COVID-19 pandemic (9, 85), or whether it was a symptomatology that the participants could be carrying over from the work routine prior to the pandemic (17). Also, the participants did not have a previous diagnosis, therefore the results should be considered provisional, and it is necessary to continue to investigate empirically the relationships between the variables. Future studies could investigate through longitudinal studies the levels of PHQ9 in teachers, in order to know with greater certainty to what extent their levels of emotional intelligence are associated.

## Practical implications

Beyond the limitations, this research provides evidence on the implications of burnout and emotional intelligence on the mental health and thus on the job performance of teachers (84). Teachers are one of the main assets in a society as they are in charge of transmitting knowledge and educating citizens (18). Therefore, the development of their emotional capabilities should be a priority. The implementation of programs that promote health in work environments, which have been shown to be an investment and not an expense, by having a positive return so, is posed as imperative. In the current global situation, every small contribution can make a big difference, so a useful solution to reduce the impact of burnout is to develop emotional skills. For this reason, training programs should aim not only to develop the emotional skills of professionals to prevent burnout problems, but also to promote individual outcomes (17). Also, this approach could help to improve the management of emotions, reducing the high impact that negative institutional and personal consequences—such as sick leave or constant rotations—have on the schools' competitiveness.

## Data availability statement

The raw data supporting the conclusions of this article will be made available by the authors, without undue reservation.

## Ethics statement

Ethical review and approval was not required for the study on human participants in accordance with the local legislation and institutional requirements. Written informed consent from the participants was not required to participate in this study in accordance with the national legislation and the institutional requirements.

## Author contributions

LS-P, TG, EE, and DN conceived the study, participated in study design, data collection, interpretation of the result, drafting the manuscript, and revised the manuscript critically. All authors contributed to the article and approved the submitted version.
